# Material Characterisation and Stratification of Conjunctival Epithelial Cells on Electrospun Poly(ε-Caprolactone) Fibres Loaded with Decellularised Tissue Matrices

**DOI:** 10.3390/pharmaceutics13030318

**Published:** 2021-02-28

**Authors:** Lucy A. Bosworth, Kyle G. Doherty, James D. Hsuan, Samuel P. Cray, Raechelle A. D’Sa, Catalina Pineda Molina, Stephen F. Badylak, Rachel L. Williams

**Affiliations:** 1 Department of Eye and Vision Science, Institute of Life Course and Medical Sciences, Faculty of Health and Life Sciences, University of Liverpool, Liverpool L7 8TX, UK; K.Doherty@liverpool.ac.uk (K.G.D.); JAMES.HSUAN@liverpoolft.nhs.uk (J.D.H.); hlscray@liverpool.ac.uk (S.P.C.); rlw@liverpool.ac.uk (R.L.W.); 2 Liverpool University Hospitals NHS Foundation Trust, Liverpool L9 7AL, UK; 3 Department of Mechanical, Materials and Aerospace Engineering, Faculty of Science and Engineering, University of Liverpool, Liverpool L69 3GH, UK; rdsa@liverpool.ac.uk; 4 McGowan Institute for Regenerative Medicine, University of Pittsburgh, Pittsburgh, PA 15219, USA; CAP131@pitt.edu (C.P.M.); badylaks@upmc.edu (S.F.B.); 5 Department of Surgery, School of Medicine, University of Pittsburgh, Pittsburgh, PA 15213, USA; 6 Department of Bioengineering, University of Pittsburgh, Pittsburgh, PA 15261, USA

**Keywords:** electrospinning, conjunctiva, decellularized tissue matrix, small intestinal submucosa, urinary bladder matrix, polycaprolactone, fiber, tissue engineering, stratification, conjunctival epithelial cells

## Abstract

The conjunctiva, an under-researched yet incredibly important tissue, plays key roles in providing protection to the eye and maintaining homeostasis of its ocular surface. Multiple diseases can impair conjunctival function leading to severe consequences that require surgical intervention. Small conjunctival defects can be repaired relatively easily, but larger defects rely on tissue grafts which generally do not provide adequate healing. A tissue engineering approach involving a biomaterial substrate capable of supporting a stratified epithelium with embedded, mucin-secreting goblet cells offers a potential solution. As a first step, this study aimed to induce stratification of human conjunctival epithelial cells cultured on electrospun scaffolds composed from poly(ε-caprolactone) (PCL) and decellularised tissue matrix (small intestinal submucosa (SIS) or urinary bladder matrix (UBM)) and held at the air/liquid interface. Stratification, up to 5 cell layers, occurred more frequently on scaffolds containing PCL + UBM. Incorporation of these decellularised tissue matrices also impacted material properties, with significant changes occurring to their fibre diameter, tensile properties, and chemical composition throughout the scaffold structure compared to PCL alone. These matrix containing scaffolds warrant further long-term investigation as a potential advanced therapy medicinal product for conjunctiva repair and regeneration.

## 1. Introduction

The conjunctiva is a mucous membrane that has an important role in maintaining a normal ocular surface and motility of the eye and eyelids. The palpebral conjunctiva lines the posterior surfaces of the eyelids and is reflected in the fornices to become the bulbar conjunctiva overlying the anterior sclera until it merges with the cornea. The total surface area is approximately 15 cm^2^ [1]. It has a stratified epithelium up to six layers thick with numerous goblet cells, and an underlying basement membrane. Beneath is the stroma, which comprises loose, vascular connective, and lymphoid tissue as well as the accessory lacrimal glands of Krause and Wolfring.

The conjunctiva serves to protect the eye and contributes to a healthy ocular surface and tear film through mucin production by the goblet cells. These not only stabilise the epithelium—tear film interface which helps to lubricate the ocular surface, but also have some antimicrobial activity [2]. A healthy conjunctiva is necessary for normal ocular motility, and has innate and adaptive immune responses to defend against pathogens [3].

Conjunctival diseases are numerous, but of greatest interest to tissue engineering are those that result in significant loss of healthy conjunctiva, which may in turn cause corneal problems with loss of vision, or double vision. These include mucous membrane pemphigoid, Stevens-Johnson syndrome, trauma including burns, malignancy, and complicated glaucoma or socket surgery. Of these, malignancy potentially offers the greatest scope for engineered conjunctival substitutes as following complete resection of a tumour there may be an extensive defect but the remaining tissue is healthy and provides a good host environment for a transplant. Other conditions may affect the whole conjunctiva or recur in the transplant [4].

**Figure 1:**
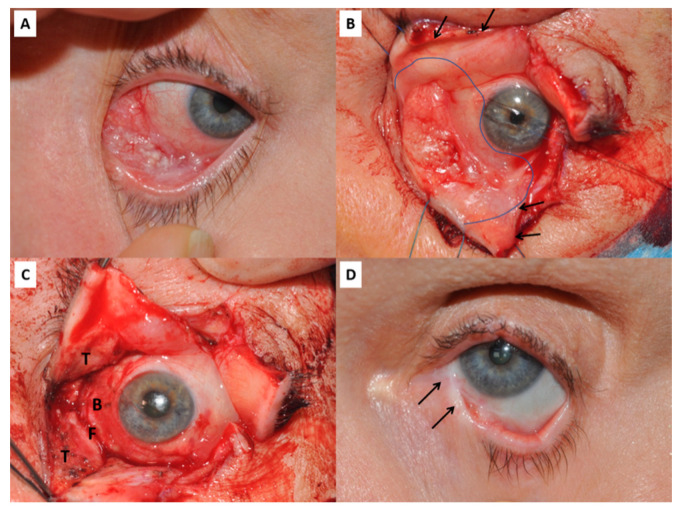
(**A**) Conjunctival squamous cell carcinoma affecting the infero-medial quadrant of the left eye. (**B**) Same patient during surgery. The upper and lower eyelids have undergone a full-thickness vertical lid-split procedure (arrows mark the cut edges) to improve visualisation and access to the tumour. The intended resection margin is shown by the curved line. (**C**) Following resection of the tumour there is an extensive conjunctival defect involving the tarsal plates and conjunctiva (T), the inferior fornix (F) and the bulbar conjunctiva (B). (**D**) Late post-operative image of the same patient showing symblepharon between the lower lid and globe (arrows), despite reconstruction with amniotic membrane, a free tarsal graft to the lower lid and temporary Gore-tex sheets as spacers.

Small conjunctival defects can be closed directly or repaired with an autologous graft, but larger defects have traditionally required either amniotic or mucous membrane grafting. In this instance, amniotic membrane acts as an inlay graft or basement membrane substitute to promote overgrowth of host conjunctival epithelial cells. However, where defects are very large or involve opposing conjunctival surfaces, there is a risk of scarring and symblepharon developing before epithelialisation has occurred, which can lead to diplopia from limited motility and lid malposition (Figure 1) [5]. Oral mucous membrane largely overcomes this problem, but is quite different to conjunctiva being much thicker and lacking goblet cells. It does not support the tear film, is cosmetically poor and can impair detection of recurrent disease. At present, local resection of very extensive conjunctival tumours is limited by the current reconstructive techniques. Tissue engineered conjunctival substitutes offer the potential to overcome this barrier, and reduce the risk of post-operative diplopia and ocular surface problems due to conjunctival deficiency.

To be clinically-usable and with demonstrable long-term positive outcome, an advanced therapy medicinal product (ATMP) for the conjunctiva needs to possess a number of essential properties, including:

- robust enough to handle with surgical instruments without becoming disrupted or compromised
- able to retain a suture for fixation
- manufactured within a limited time frame
- non-allergenic and provoking minimal inflammatory/scarring response
- have an intact epithelium sufficient to prevent symblepharon/adhesions, and which allows eventual transition to a normal conjunctival epithelium including goblet cells
- a sufficiently mobile stroma to allow full extraocular movements

These criteria build on those previously outlined by Schrader et al. [6], who also commented on a conjunctival ATMP needing to mimic the natural tissue architecture and possess sufficient elasticity to prevent or reduce contraction. As discussed in several detailed reviews, a number of biological and synthetic materials have been researched and developed with the aim of creating the optimal ATMP for *ex vivo* culture of conjunctival epithelial and goblet cells, which, following transplantation, should lead to repair and regeneration of the conjunctiva [6,7,8]. However, whilst biological or synthetic materials are frequently selected due to their known key benefits, they often possess other less favourable properties that can limit their success as an ATMP.

In recent years, research in tissue decellularisation has significantly increased and there are multiple decellularised tissue products that are commercially available [9]. Decellularisation of mammalian tissues and organs involves the complete removal of cells to leave the tissue’s 3D structural architecture that may then be re-populated with a different source of cells (e.g., allogeneic) to create new, engineered tissues. With application of complex, multi-step decellularisation protocols to efficiently eliminate all cellular antigens, the remaining extracellular matrix (ECM) structure should provide a near-perfect blueprint of appropriate dimensional scale and ideal combination of biomolecules (i.e., proteins and polysaccharides) to support cell adhesion, proliferation and, importantly, phenotype and differentiation [10]. Yet, as described in the detailed review by Gilbert et al. [11], the need to completely remove all cellular components requires both physical (e.g., sonication, snap-freezing, mechanical force) and chemical (e.g., enzymatic digestion, ionic solutions, detergents) processing, which can adversely affect the tissue’s natural structure and hence impact its resulting biological and mechanical properties. Changes to the tissue’s microstructure may impede its mechanical and functional properties, resulting in a fragility that makes it difficult to handle without incurring further damage [12,13,14]. To overcome this, it is possible to mill lyophilised decellularised tissue (dECM) into a powder, retaining its unique cocktail of biological properties, and subsequently form a hydrogel via its solubilisation and neutralisation, as described by Saldin et al. [15]. 3D dECM-hydrogels support the encapsulation of viable cells and overcome the difficulties associated with successfully recellularising bulk decellularised tissues [16]. The development of dECM powders used to create these biological hydrogels is an active area of research and have been sourced from a wide variety of tissues and whole organ systems, including (but not limited to) musculoskeletal, cardiac, liver, kidney, and skin. Porcine-derived small intestinal submucosa (SIS) and urinary bladder matrix (UBM) are two other tissues commonly used to create dECM hydrogels. Not surprisingly, the different anatomical locations of these two tissues renders differences in their structural, functional and biochemical properties. For example, SIS-matrices comprise approximately 90% collagen, being predominantly type I with minor quantities of types III, IV, V, and VI, whereas UBM-matrices contain almost identical collagen types, but with a greater quantity of type III and the addition of type VII, which are essential components of epithelial basement membranes [17].

Whilst exploration of these dECM hydrogels continues in research groups worldwide, they remain mechanically weak which can restrict their clinical usability [18]. These mechanical changes may be overcome by incorporating the dECM powder with other, more durable biomaterial substrates, such as electrospun fibres. Electrospinning is a popular technique for fabricating fibre scaffolds that mimic the structural properties of tissues [19]. This approach therefore offers several advantages: the fibrous substrate confers structural support and topographical cues, and the powdered dECM provides the cells with a specific mix of biomolecules known to support and maintain that particular cell phenotype [20,21].

In this study, decellularised tissue powders (SIS or UBM) with poly(ε-caprolactone) (PCL; a biodegradable, synthetic polymer) were electrospun to create bioactive, fibre scaffolds with the aim of developing a novel conjunctival ATMP. Research findings demonstrated dECM-containing PCL scaffolds exhibited notable differences both in terms of material properties (fibre morphology, mechanical) and in vitro cell response (cell morphology, stratification) compared to PCL alone.

## 2. Materials and Methods

### 2.1. Tissue Decellularisation

UBM and SIS scaffolds were prepared at the University of Pittsburgh, from porcine tissue sources at Animal Biotech Industries (Doylestown, PA, USA), as outlined in Keane et al. [22]. Briefly, for UBM preparation, the urethra and ureter were removed from the bladders. Bladders were opened along their length and mechanically scraped to remove the tunica serosa, tunica muscularis externa, tunica submucosa, and tunica muscularis mucosa. Further, rinsing with deionised water was used to remove the urothelial cells on the surface of the tunica mucosa. For SIS preparation, intestines were flushed with distilled water, opened along their length and mechanically scraped to remove the tunica mucosa, tunica serosa, and tunica muscularis externa. Decellularisation of the remaining tissues was performed using a solution of 0.1% *v*/*v* peracetic acid (Rochester Midland Corporation, Rochester, NY, USA) and 4% *v*/*v* ethanol (200 proof; Decon Laboratories Inc., King of Prussia, PA, USA) in type I water (Thermo Scientific, Waltham, MA, USA), with agitation at 300 rpm in a shaker, for 2 h. The resulting UBM and SIS were then washed three times, alternating between phosphate buffered saline (PBS; Fisher BioReagents, Pittsburgh, PA, USA) and sterile water, for 15 min on an orbital shaker at 300 rpm each time. The UBM and SIS scaffolds were lyophilised and milled into powder using a #60 mesh screen on a Wiley Mill (GE Motors & Industrial Systems, Houston, TX, USA) [23].

### 2.2. Solution Preparation

Powdered decellularised porcine tissues (small intestinal submucosa (SIS) and urinary bladder matrix (UBM)) were received at the University of Liverpool. Solutions of poly(ε-caprolactone) (PCL; Purasorb PC12, Corbion, Amsterdam, The Netherlands) dissolved in 1,1,1,3,3,3-hexafluoroisopropanol (HFIP; Merck, Gillingham, UK) were prepared at concentrations of 12%w/v plus 1% or 10% SIS or UBM and stirred continuously for 48 h at room temperature.

### 2.3. Scaffold Fabrication

Solutions were loaded into separate capillary-ended syringes with an applied flow rate of 1 mL/h and subsequently electrospun (50 min per run) using an IME Technologies EC-CLI unit with controlled ambient environment (temperature 21 °C, relative humidity 50%) and applied spinning parameters: needle voltage +15 kV, collector voltage −4 kV, and distance 17 cm. Emitted fibres were collected on a mandrel lined with wax paper (PME, Enfield, UK) and rotating at 100 rpm.

### 2.4. Material Characterisation

#### 2.4.1. Fibre Morphology and Diameter

Scanning electron microscopy (SEM) was used to obtain high magnification images of the fibre scaffolds. Scaffolds were mounted on carbon-tabbed SEM stubs (Agar Scientific Ltd., Stansted, UK) and AuPd sputter-coated for an even coverage. Scaffolds were imaged using a FEI Quanta 250 FEG SEM operating at high vacuum with 5 kV electron beam.

Fibre diameters were measured by analysing the SEM images using ImageJ software (v.1.53c, National Institutes of Health, Bethesda, MD, USA). This was achieved by using the line draw tool and the scale bar of each image to initially set the scale. Measured diameters were statistically analysed using GraphPad Prism v9 (San Diego, CA, USA). Fibre diameters for each group (*N* = 3; *n* = 900) were not normally distributed and were subsequently analysed using a Kruskal–Wallis test with Dunn’s multiple comparisons test. Data are presented as the median and interquartile range.

#### 2.4.2. Tensile Testing

For tensile testing, fibre scaffolds were cut into 3 × 1 cm rectangles and placed and secured (using sticky tape) over a paper window to give final test dimensions of 2 × 1 cm. Scaffold thickness was measured using an electronic micrometer. Paper windows allowed easy handling and positioning of the scaffold within tensile grips. Window sides were cut prior to commencement of the tensile test to ensure only the fibre scaffold was loaded. A UniVert (CellScale; Waterloo, ON Canada) in tensile mode was used with 1 N load cell and 10% strain (*n* = 6). Data were processed in MS Excel and Graphpad Prism (v.9, GraphPad Software, Inc., San Diego, CA, USA), where a one-way ANOVA with Tukey’s multiple comparisons test was applied.

#### 2.4.3. Chemical Spectroscopy (FTIR, Imaging-Mass Spectrometry)

Chemical composition of the scaffolds was initially determined using a Fourier transform infra-red spectrometer (Bruker Vertex 70v; Bruker, Durham, UK) with diamond attenuated total reflection. A background scan was taken prior to scaffold analysis and subsequently subtracted from that scaffold’s spectrum. The scaffold was pressed into close contact with the diamond and the spectrum measured. Data were obtained in the range of 450–4000 cm^−1^, with 4 cm^−1^ resolution and scan number 32. Data were analysed with OPUS software (v8.2.28; Bruker, Durham, UK).

For imaging-mass spectrometry, data were collected on a Waters Synapt G2-Si in MALDI using HD Imaging 1.4 (Waters UK, Elstree, UK) to set acquisition parameters and MassLynx 4.1 SCN9509 (Agilent, Stockport, UK) to process the data for PCL, PCL + SIS10% and PCL + UBM10%. Acquisition parameters were set to collect masses over the 100–2000 mass/charge (*m/z*) range in positive resolution mode. Scan time per pixel was 0.5 s at 250 Laser Energy and a repetition rate of 500 Hz. An area of 14 mm^2^ was collected with a pixel size of 50 × 50 µm.

### 2.5. In Vitro Set-Up and Cell Culture

A human conjunctival epithelial cell (HCjE) line [24] was cultured at 37 °C and 5% CO_2_ in 75 cm^2^ sterile flasks (Greiner Bio-one, Stonehouse, UK) with keratinocyte serum-free medium (KSFM; Thermo Fisher, Loughborough, UK) supplemented with 25 µg/mL bovine pituitary extract (Thermo Fisher), 0.4 mM calcium chloride (Merck, Gillingham, UK), 0.2 ng/mL recombinant human epidermal growth factor (rEGF, Thermo Fisher, Loughborough, UK), 100 U penicillin and 0.1 mg/mL streptomycin (Merck, Gillingham, UK) and 2.5 µg/mL amphotericin B (Merck, Gillingham, UK).

Within a sterile, laminar flow cabinet the membranes of 24-well transwells (Millicell; Merck, Gillingham, UK) were removed and replaced with the fibre scaffolds. Scaffolds were secured by using silicone glue (Dowsil 732 (clear); Dow Corning, Penarth, UK), which was limited to the side of the transwell and not present within the new fibrous base of the transwell. Transwells were irradiated with ultraviolet light for 30 min on both sides and then disinfected by submerging in 70% *v/v* Ethanol (VWR, Lutterworth, UK) overnight. Ethanol was removed and transwells washed twice in sterile phosphate buffered saline solution (PBS; Thermo Fisher, Loughborough, UK) before being transferred to new, sterile 24-well plates (Greiner Bio-One, Stonehouse, UK).

**Figure 2:**
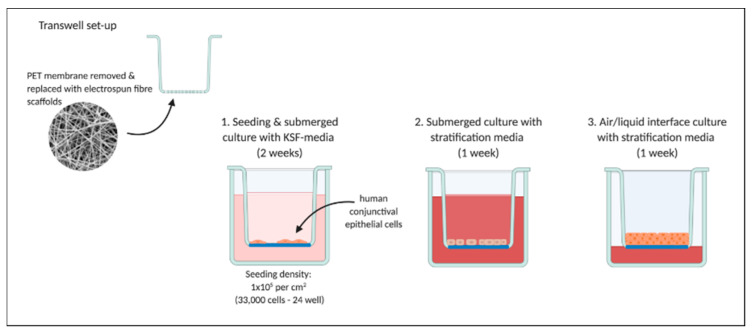
Experimental set-up for in vitro culture of human conjunctival epithelial cells demonstrating preparation of transwells with removal of polyester (PET) membrane and replacement with electrospun scaffolds. Cells were seeded and cultured fully submerged for two weeks with keratinocyte serum free media (KSF-media), followed by one week with stratification media and a further week at the air/liquid interface with media received on the cells’ basal side. Created with BioRender.com.

A density of 1 × 10^5^ cells per cm^2^ were seeded directly onto each scaffold and after 30 min topped-up with KSFM. After 14 days of growth, samples were switched to stratification media as outlined in Gipson et al. [24]. Stratification media was composed of: Dulbeccos modified Eagles media/Ham’s nutrient buffer F-12 (DMEM/F12 1:1; Merck, Gillingham, UK), 10% foetal calf serum (Biosera, Nuaille, France), 10 ng/mL rEGF, 100 U penicillin and 0.1 mg/mL streptomycin, and 2.5 µg/mL amphotericin B. Following 7 days in stratification media cultures were switched to air/liquid interface, where media was removed from the apical chamber and received on the basal-side only to further promote stratification over an extra 7 days. Figure 2 provides a schematic of the in vitro experimental set-up.

### 2.6. In Vitro Characterisation

#### 2.6.1. Assessing Cell Morphology by SEM

Cell-seeded scaffolds were washed in sterile PBS and fixed in 1.5% *v/v* glutaraldehyde (TAAB Laboratories, Aldermaston, UK) in PBS for 30 min at 4 °C. Scaffolds (*n* = 2) were subsequently dehydrated in increasing concentrations of Ethanol; 50, 70 and 90% *v/v* for 2 × 3 min and 100% *v/v* for 2 × 5 min. Finally, scaffolds were chemically dried in hexamethyldisilazane (Sigma, UK) for 2 × 5 min. Scaffolds were mounted on carbon-tabbed SEM stubs, AuPd sputter-coated, and imaged on a Hitachi TM4000 Plus (Hitachi, Warrington, UK) at 15 kV in BSE mode and high vacuum. 

#### 2.6.2. Assessing Cell Stratification by Confocal Microscopy

Cell-seeded scaffolds were washed in sterile PBS and fixed in 10% neutral buffered formalin (Merck, Gillingham, UK) for 10 min at room temperature. Samples were washed several times in PBS, permeabilised with 0.5% Triton-X for 5 min and washed three times in PBS. DAPI (1:1000; Thermo Fisher, Loughborough, UK) was applied for 20 min at room temperature (in the dark) to stain the cells’ nuclei. Scaffolds were washed in PBS and subsequently mounted on glass microscope slides. Substrates were imaged by confocal microscopy (Zeiss LSM800; Cambridge, UK) at ×40 magnification and images processed using Fiji ImageJ (v.1.53c, National Institutes of Health, Bethesda, MD, USA). Three set coordinates within the XY image were randomly selected and applied to images for all scaffold groups. This generated 6 regions of interest (area = 298.46 × 40.80 μm) per sample (*n* = 3) that were viewed in the orthogonal slice: 3 in the XZ plane (co-ordinates: 190, 256, 500) and 3 in the YZ plane (co-ordinates: 180, 256, 400). Distinctly separated nuclei were counted and instances of cell nuclei in close proximity and stacking on top of each other noted, for example, 3 nuclei stacked on top of each other would be 3 cells counted and 1 instance of a triple cell layer. The number of instances, where cells were present either as a monolayer or 2-, 3-, 4-, 5-layers, were totalled and the proportion for each type of layer presented as a percentage of this total. The labelling of images was removed prior to processing to blind the operator and remove the potential for bias. Data were processed in Graphpad Prism v.9 (San Diego, CA, USA) and a one-way ANOVA with Tukey’s multiple comparisons test applied for the total cell number within each group (*n* = 3).

## 3. Results

### 3.1. Material Characterisation

#### 3.1.1. Fibre Morphology and Diameter

**Figure 3:**
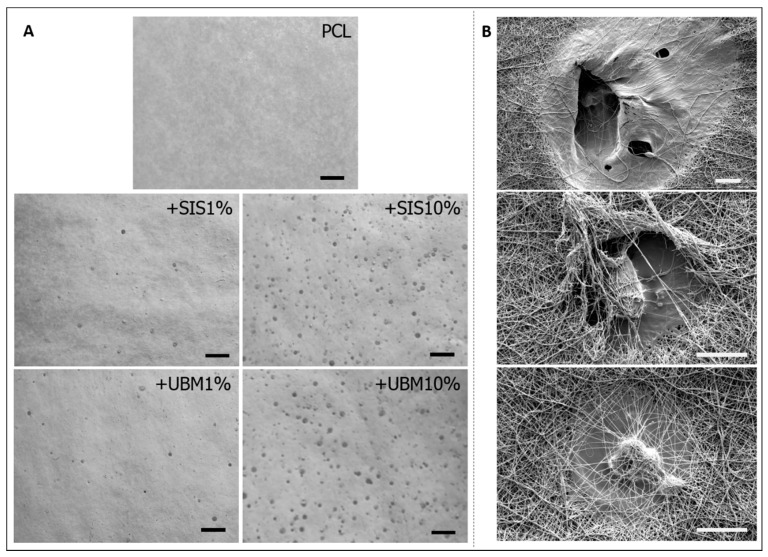
(**A**) Gross images of as-spun electrospun fibres for poly(ε-caprolactone) (PCL) and with the addition of 1% or 10% decellularised tissue powder (dECM—small intestinal submucosa (SIS) and urinary bladder matrix (UBM)) (scale = 5 mm). Images converted to greyscale for observation of ‘wet’ droplets on dECM containing fibre scaffolds. (**B**) Scanning electron microscopy images highlighting the ‘wet’ droplets present within the PCL + SIS10% fibres and the holes these create. Scale bar = 40 μm, magnification ×1000 (top image) and ×2000 (middle and bottom images).

There were no observable issues during the electrospinning process. The rotating mandrel, which was used to target and collect the emitted polymeric jet following its propulsion across the air-gap, was evenly covered following a 50-min spin time for each of the five solutions that were electrospun. Gross inspection of the collected fibres revealed differences between the groups (Figure 3A). Electrospun PCL presented uniform coverage across the sheet of wax paper (used to easily remove collected fibres for analysis), with an even white colour and smooth texture. The inclusion of decellularised tissue powders (dECM) to the PCL, however, demonstrated numerous droplets throughout the collected area, which increased in number with greater dECM content. Inspection by SEM revealed these droplets to have dried on top and within the surrounding fibres to create areas of flattened topography (Figure 3B). Removal of fibres did, on occasion, result in tearing of the scaffold where droplets had contacted and dried to the wax-backing paper. This only occurred in scaffolds containing larger quantities of dECM.

**Figure 4:**
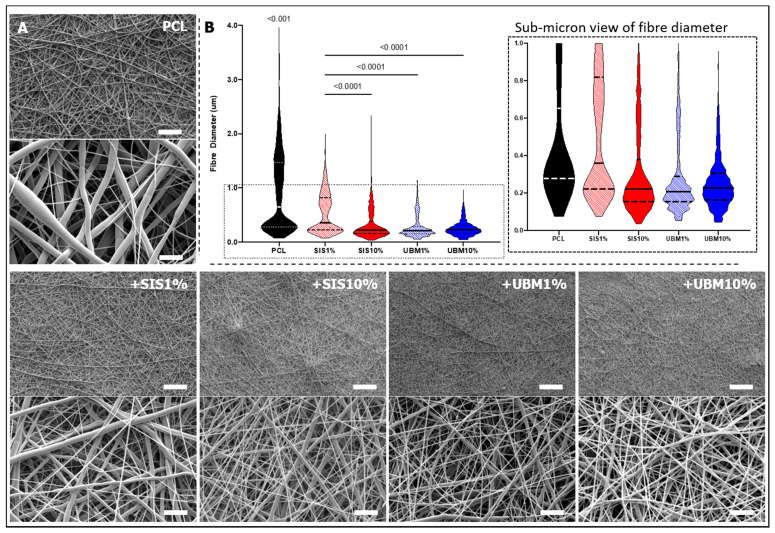
(**A**) Scanning electron microscopy images of electrospun poly(ε-caprolactone) (PCL) scaffolds and following the addition of 1% or 10% decellularised tissue powder (small intestinal submucosa (SIS) and urinary bladder matrix (UBM)). Low magnification images ×1000, scale = 50 μm; high magnification images ×10,000, scale = 5 μm. (**B**) Measured fibre diameter presented as a violin plot for each group with magnified view of data spread within the sub-micron range (*n* = 900). Kruskal–Wallis statistical test with Dunn’s multiple comparisons (*p* < 0.05). PCL was significantly different in all groups.

Separate to these droplets, the bulk of polymer deposition on the collector sheet revealed fibres with rounded and smooth morphologies (Figure 4). High magnification SEM images revealed a shift in fibre morphology, where PCL alone demonstrated two distinct fibre sizes, but the inclusion of dECM resulted in the fabrication of finer and more uniform fibres. This was particularly noticeable for PCL + UBM10%. Measurement of fibre diameter for all groups supported these visual findings, with fibres in all groups containing dECM being significantly finer than pure PCL fibres. PCL fibres possessed a median fibre diameter of 0.65 μm (IQR 0.28–1.46 μm). Addition of 1% dECM powder, resulted in a notable reduction, with median fibre diameters measuring 0.36 μm (IQR 0.22–0.82 μm) upon addition of SIS and 0.21 μm (IQR 0.15–0.29 μm) for UBM. Increasing the quantity of dECM to 10% demonstrated a further decrease in fibre diameter for SIS (median 0.22 μm (IQR 0.15–0.38 μm)), but a similar distribution of fibre diameters for UBM (median 0.22 μm (IQR 0.16–0.31 μm)). UBM10% was the only group to produce a fibre range below 1 μm.

#### 3.1.2. Chemical Spectroscopy (FTIR and Imaging-Mass Spectrometry)

**Figure 5:**
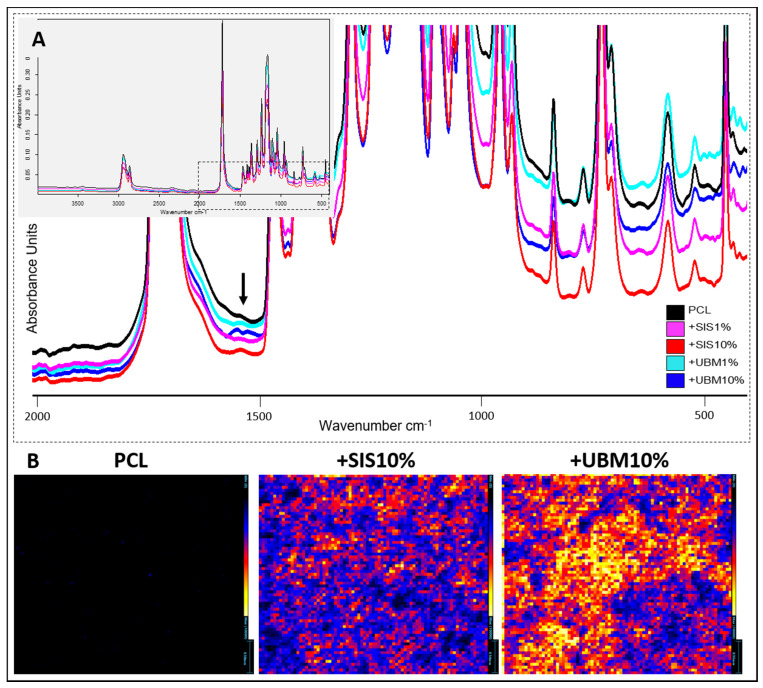
Spectroscopic analysis of electrospun poly(ε-caprolactone) (PCL) scaffolds and following the addition of decellularised tissue powder (small intestinal submucosa (SIS) and urinary bladder matrix (UBM)). (**A**) Fourier transform infra-red spectroscopy with inset demonstrating complete spectra for each group, and magnified view of chemical change at 1590 cm^−1^ representative of a primary amine (N-H bend), which was most noticeable for SIS10% and UBM10% groups. (**B**) Imaging-mass spectrometry demonstrating greater ionisation (and even distribution) in SIS10% and UBM10% groups compared to PCL at *m/z* 523. Maximum number of counts = 10,000. Scale for PCL = 0.58 mm, SIS10% = 0.54 mm, UBM10% = 0.55 mm.

In order to detect the presence of decellularised tissue within the PCL fibres, FTIR and Imaging-Mass Spectrometry were performed. Comparison of the scaffolds’ complete spectra revealed no obvious differences (Figure 5A). Characteristic chemical bonds for esters were identified C=O (1750–1735 cm^−1^) and C–O (1260–1000 cm^−1^), in addition to C–H bonds (2960–2850 cm^−1^). Closer examination demonstrated a distinct peak within 1650–1590 cm^−1^ which was most apparent for PCL + SIS10% and PCL + UBM10%.

Imaging-mass spectrometry (Imaging-MS) allowed the spatial distribution of molecular species to be visualised between PCL and those containing the greatest quantity of dECM powder (Figure 5B). Normalisation of the data to 10,000 counts demonstrated clear differences in the heat map for each group. Minimal ionisation was detected across the surface for PCL fibres, but this increased with inclusion of 10%SIS, where low to mid counts (~5000) were detected, and increasing further from low to high counts (~10,000) for 10%UBM. Presence of UBM and SIS increased the ionisation of the fibres at several mass-to-charge (*m/z*) values, including a *m/z* peak at 523.25. The imaging element of this technique revealed these increased intensities to be distributed throughout the fibre scaffolds.

#### 3.1.3. Tensile Properties

**Figure 6:**
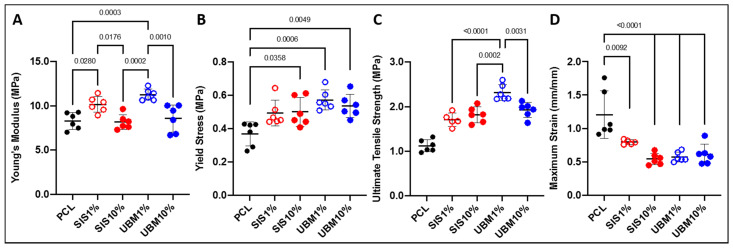
Tensile testing data for scaffolds of electrospun poly(ε-caprolactone) (PCL) and with the addition of 1% or 10% decellularised tissue powder (small intestinal submucosa (SIS) and urinary bladder matrix (UBM)): (**A**) Young’s modulus, (**B**) yield stress, (**C**) ultimate tensile strength and (**D**) maximum strain at break. One-way ANOVA with Tukey’s multiple comparisons (*n* = 6), significance for *p* < 0.05. Statistical differences shown by *p* values.

Scaffolds were subjected to mechanical testing to determine any change in their tensile properties following addition of dECM (Figure 6). In terms of Young’s modulus, electrospun PCL scaffolds yielded a stiffness of 8.29 ± 0.94 MPa (Figure 6A). The inclusion of 1% dECM resulted in a significant increase in scaffold stiffness compared to PCL alone, where SIS1% and UBM1% were 10.16 ± 0.89 MPa (*p* = 0.0280) and 11.25 ± 0.64 MPa (*p* = 0.0003), respectively. However, a similar trend was not observed for either 10% dECM groups, with Young’s modulus calculated as 8.17 ± 0.84 MPa (SIS10%) and 8.58 ± 1.53 MPa (UBM10%). The yield stress (i.e., the maximum tensile load before plastic deformation) of PCL was 0.37 ± 0.07 MPa (Figure 6B). The addition of dECM powders revealed a general increase in the maximum load that the scaffolds were able to withstand. This was particularly evident for UBM-containing scaffolds with UBM1% yielding at 0.57 ± 0.06 MPa (*p* = 0.0006) and UBM10% at 0.54 ± 0.07 MPa (*p* = 0.0049). Blends of PCL and SIS resulted in yield stresses of 0.49 ± 0.08 MPa (not significant) and 0.50 ± 0.09 MPa (*p* = 0.036) for 1% and 10% dECM, respectively. The ultimate tensile strength (UTS) of PCL scaffolds was 1.12 ± 0.14 MPa (Figure 6C). All scaffolds containing dECM had a significantly greater UTS compared to PCL (*p* < 0.0001). Inclusion of SIS increased the UTS to 1.70 ± 0.15 MPa and 1.82 ± 0.18 MPa for 1% and 10%, respectively. UBM1% had the highest UTS overall (2.31 ± 0.18 MPa) and was significantly different to all other scaffolds (*p* < 0.0001 vs. SIS1%, *p* = 0.0002 vs. SIS10%, and *p* = 0.0031 vs. UBM10%). UBM10% scaffolds had a UTS of 1.92 ± 0.17 MPa. The maximum strain reached at the UTS also presented significant decreases following dECM blending (Figure 6D). Maximum strain for PCL scaffolds was 1.21 ± 0.36 mm/mm. For SIS1% and SIS10%, scaffold extension reduced to 0.80 ± 0.03 mm/mm (*p* = 0.0092) and 0.54 ± 0.08 mm/mm (*p* < 0.0001), respectively. Similarly, UBM1% and UBM10% yielded strains of 0.57 ± 0.07 mm/mm (*p* < 0.0001) and 0.61 ± 0.15 mm/mm (*p* < 0.0001).

### 3.2. Air/Liquid Interface Culture and Stratification

**Figure 7:**
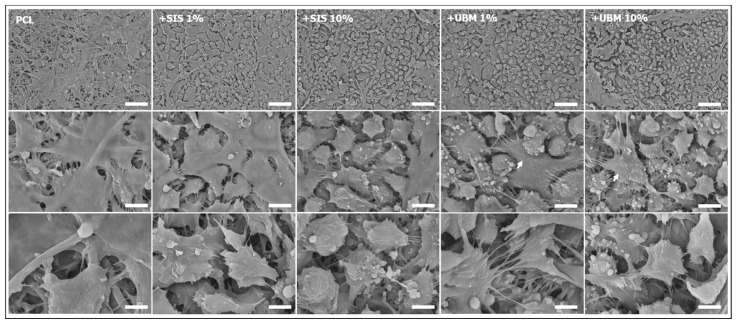
Representative scanning electron microscopy images of human conjunctival epithelial cells cultured on electrospun scaffolds fabricated from poly(ε-caprolactone) (PCL) and PCL with addition of 1% or 10% decellularised tissue powder (small intestinal submucosa (SIS) and urinary bladder matrix (UBM)). Images: top row = ×1000 (scale = 50 μm), middle row = ×5000 (scale = 10 μm), bottom row = ×10,000 (scale = 5 μm); arrows indicate presence of microvilli.

**Figure 8:**
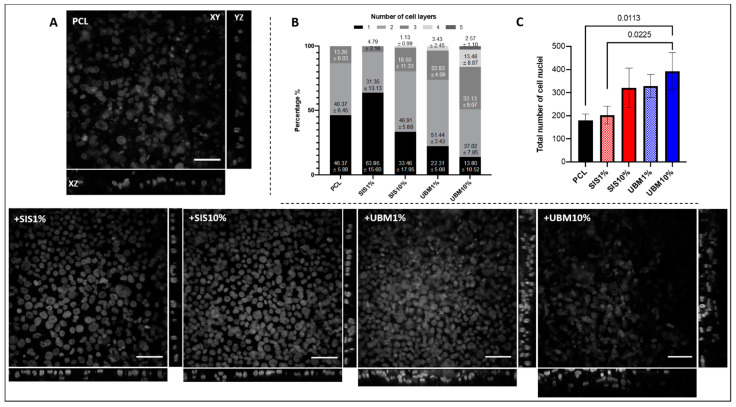
(**A**) Representative confocal images of DAPI-stained human conjunctival epithelial cell nuclei cultured on electrospun scaffolds fabricated from poly(ε-caprolactone) (PCL) and PCL with addition of 1% or 10% decellularised tissue powder (small intestinal submucosa (SIS) and urinary bladder matrix (UBM)). Images shown; XY z-stack (scale = 50 μm), XZ side view and YZ side view. (**B**) Cell layers observed from 6 set regions of interest presented as percentages within each group (*n* = 3). (**C**) Total number of cell nuclei counted within 6 regions of interest for each group (*n* = 3). One-way ANOVA with Tukey’s multiple comparisons, significance for *p* < 0.05. Statistical differences shown by *p* values.

The response of human conjunctival epithelial cells (HCjE), in terms of their ability to stratify on the different scaffolds was investigated. HCjE cells were cultured on the scaffolds for a total of four weeks, where the final week was at the air/liquid interface. SEM imaging revealed a difference in cell response to the fibre scaffolds (Figure 7). Low magnification (×1000) images for the PCL scaffold revealed patches of electrospun fibres with limited presence of cells. In contrast, dECM-containing scaffolds demonstrated complete cell coverage over the fibre surface at the same magnification. HCjE cells on PCL-only scaffolds appeared well spread with flattened morphologies. In some regions, it was possible to view the shape of fibres situated beneath the cells. Cellular processes enabling cell-to-cell contact were evident. Indication of HCjE stratification on PCL fibres appeared limited. Observation through the z-plane of PCL scaffolds and DAPI-stained cell nuclei by confocal microscopy revealed occasions of two (40.47 ± 6.45%) and three (13.26 ± 6.03%) cell nuclei loosely-stacked on top of each other (Figure 8A,B). Single nuclei accounted for 46.37 ± 5.88% and the total number of nuclei (180 ± 28) counted from the six regions of interest (*n* = 3) was lowest on PCL scaffolds overall (Figure 8B,C). SEM images for dECM-containing scaffolds identified two main morphologies: small, rounded cells with multiple cellular processes, and larger, elongated cells with flattened appearances (Figure 7). These flattened cells appeared in greater numbers within the SIS1% cohort. A breakdown of cell layers for SIS1% from the total number of counted nuclei (202 ± 39) revealed single cells accounting for 63.86 ± 15.60%, two-layers 31.35 ± 13.13% and three stacked-nuclei occurring 4.79 ± 2.16%. A greater number of cell nuclei (321 ± 85) were counted in the SIS10% group, with observed instances of one, two, three and even four-layers from the regions of interest being 33.46 ± 17.95%, 46.91 ± 5.88%, 18.50 ± 11.33% and 1.13 ± 0.99%, respectively. UBM-containing groups supported superior stratification with greater instances of four and occasionally five layers of cell nuclei evident in the regions of interest. For UBM1% 329 ± 50 cell nuclei were counted, with 22.31 ± 5.08% being single nuclei, 51.44 ± 3.43% two nuclei, 22.82 ± 4.09% three nuclei and 3.43 ± 2.45% four-stacked nuclei. For UBM10%, from 392 ± 81 counted nuclei, 13.80 ± 10.52% were single, 37.02 ± 7.85% two-layers, 33.13 ± 9.07% three-layers, 13.48 ± 8.87% four-layers, and 2.57 ± 1.10% five-layers. 

## 4. Discussion

This study investigated the incorporation of dECM powders with PCL to fabricate bioactive fibre scaffolds. The prepared solutions all yielded fibres when electrospun using the same parameters. Examination of the collected fibre sheets demonstrated macroscopic differences upon the addition of dECM to the PCL solution (Figure 3A). Instead of a uniform coverage of fibres, droplets spread randomly throughout the deposited area were visible, and these increased in number with greater dECM quantity. SEM imaging revealed these droplets to present flat/wet-looking regions located within a network of otherwise smooth, bead-free fibres (Figure 3B). Instability of the polymer jet during electrospinning can result in the formation of “beads-on-a-string”, though this was not evident from these SEM images. Yet, this phenomenon does suggest periods of jet instability giving rise to occasional electrospraying as opposed to continuous electrospinning. Another theory could be non-solubilised dECM components accelerating along the emitted polymer jet before contacting with the collector system. Inspection of the polymer/dECM solutions did reveal a granular sediment, suggesting the dECM had not fully dissolved in the solvent. To our knowledge, this presentation of droplets has not been previously reported in the literature and our findings are in contrast with studies that suggest the direct dissolution of dECM in solvent is sufficient [25,26,27,28]. Other studies initially solubilise the dECM powder in solutions of acetic acid and/or pepsin prior to dissolution with the synthetic polymer and solvent [29,30,31]. Although a study by Stankus et al. [20] does suggest that dECM agglomerates may contain proteins that remain insoluble in the selected electrospinning solvent. It is worth noting that we were later able to achieve droplet-free, uniform fibre sheets following the initial dissolution of dECM in 0.5M acetic acid and pepsin at a 1:10 with dECM (Figure S1).

Continuing with direct addition of dECM powders with PCL in solution, a significant decrease (*p* < 0.0001) in fibre diameter was determined following its incorporation (Figure 4). This was achieved irrespective of tissue source and quantity included. However, further statistically different reductions in diameter were achieved when comparing SIS1% to SIS10% and to both UBM groups. For SIS1%, the fibres presented a more bimodal distribution compared to all other dECM-containing groups with an IQR of 0.22–0.82 μm and range of 1.92 μm. As indicated in the violin plot, the majority of dECM-containing fibres measured below 1 μm, though only UBM10% yielded a fibrous network that was completely submicron and with tightest range (0.91 μm). This is considerably different to PCL which had a range of 3.88 μm. Comparison of these findings to the literature is mixed. A couple of articles (using the same method of fabrication) state no quantified change in fibre diameter following dECM inclusion [26,27], whereas Kim et al. [32] reported a significant reduction in fibre diameter (45%) following the inclusion of 1% SIS with poly(lactic-*co*-glycolic) acid (PLGA). Hong et al. [25] attributed these decreases to the elevated conductivity of solutions following addition of dECM. Solution conductivity is a known parameter that influences fibre formation during electrospinning. Solutions of higher conductivity possess greater charge and upon application of a high voltage, the emitted polymeric jet undergoes considerable stretching and thinning due to the charge repulsion, resulting in the formation of finer diameter fibres [33].

Spectroscopic analysis is a routine way to locate the presence of molecular species and FTIR and Imaging-MS were undertaken in this study. Comparison of molecular spectra by FTIR revealed no change to the chemical groups of PCL but also the identity of a new peak in dECM-containing scaffolds (Figure 5A). For SIS10% and UBM10%, a new peak was observed in the region 1650–1590 cm^−1^, which is associated with a primary amine (specifically NH bend) [34]. Imaging-MS demonstrated clear changes in scaffold composition following inclusion of dECM, which were distributed throughout the fibrous network (Figure 5B). Intensities were 100x greater than PCL alone and more notable for UBM over SIS, suggesting the slight differences in the make-up of these tissues had a direct impact on the ionisation of the scaffold. In order to ascertain what these changes may be ascribed to, Raman spectroscopy, which allows whole molecule vibrations to be analysed, is required.

When designing an ATMP for tissue repair and regeneration, the mechanical properties also need to be considered. Whilst not a load-bearing tissue per se, ATMPs for conjunctival replacement still need to possess physical characteristics sufficient enough to enable its handling and transplantation during surgery, and resist tearing when secured in place by sutures. Furthermore, a transplanted ATMP will need to be sufficiently elastic in order to support eye movements and blinking [35]. The addition of dECM powders to electrospun PCL scaffolds resulted in significant increases in Young’s modulus, yield stress and UTS, but significant decreases in maximum strain. Our findings are in general agreement with the literature, where increases in modulus [25,31,32,36], yield strength [26,37] and UTS [32], and decrease in strain [31] have also been reported. However, several other studies demonstrate different findings: Stankus et al. [20] described a linear decrease in modulus and UTS with increasing mass of UBM to poly(ester-urethane) urea; Fernandez-Perez et al. [28] found addition of decellularised cornea with PCL had no impact on scaffold stiffness; Hong et al. [29] determined increasing quantities of dECM yielded a general decrease in UTS but scaffolds were stiffer. Separate to the addition of dECM, fibre diameter is known to have a direct impact on tensile properties. A study by Wong et al. [38] demonstrated an abrupt shift in the stiffness and strength of electrospun PCL fibres at ~0.7 μm, with these properties increasing with decreasing fibre diameter. PCL fibres in this study presented a median diameter of 0.65 μm (IQR 0.28–1.46 μm), which were significantly larger than dECM-containing PCL fibres and hence likely contributed to the shift observed in the obtained tensile properties. Of further note, the Young’s modulus for both SIS10% and UBM10% did not yield significant increases as per their 1% counterparts. This can most likely be attributed to the deposition of droplets during the electrospinning process. Unfortunately, the removal of these scaffolds from the wax paper was very difficult as some of these droplets fused to the paper during drying and were unable to be removed without creating small holes in the scaffold (Figure 3B). Consequently, these defects will have contributed to the lower tensile performance of these 10% dECM scaffolds. Comparison of tensile properties for these groups to conjunctiva tissue reveals a general mismatch as the tensile strength and stiffness of human conjunctiva was reported as 0.7 MPa and 3.9 MPa, respectively [14]. Whilst these electrospun scaffolds may be considerably stronger and stiffer, further comparison to human amniotic membrane (a graft for conjunctiva) demonstrates their similarity—amnion UTS and modulus being 1.7 MPa and 11.5 MPa, respectively [14], which would suggest their general suitability.

An immortalised cell line of human conjunctival epithelial cells was cultured on these different scaffold groups [24]. This involved submerged culture for the first three weeks, with the initial two-week period using keratinocyte serum-free media and the third week switching to stratification media. At the start of the fourth week, scaffolds were cultured at the air/liquid interface, where stratification media was received by the cells on their basal side only (Figure 2). Culture at the air/liquid interface provides a better mimic of the natural in vivo environment and has been proven to promote cell proliferation, stratification and differentiation of epithelial cells [39]. Despite being cultured for the same period of time different cell morphologies were apparent between PCL and dECM-containing scaffolds (Figure 7). Low magnification SEM images revealed PCL-only scaffolds to be sparsely populated by HCjE cells despite a four-week period in culture. This is in contrast to dECM-containing scaffolds where cells had fully covered the available fibre surface. Inclusion of SIS and UBM in polyester electrospun scaffolds has previously been shown to increase cell attachment [20,32]. Furthermore, cells on PCL scaffolds appeared thinly spread and with flattened morphologies. Whilst a two-dimensional technique, it is possible to gauge a sense of depth from SEM images and for PCL scaffolds these images presented limited evidence of cell layering suggestive of stratification. Comparison to side profiles of nuclei-stained confocal images presented a slightly different view (Figure 8A). Whilst the majority of nuclei counted from the six regions of interest were located as single cells (46.37 ± 5.88%), there were many instances of two (40.37 ± 6.45%) and even three (13.26 ± 6.03%) layers of cell nuclei located within close proximity to each other (Figure 8B). However, the number of cell nuclei totalled from these regions was lowest out of the five scaffold groups and was significantly different to UBM10% (cohort with greatest number of nuclei) with a difference of 74.12% (Figure 8C). Without surface treatment, PCL alone is not an ideal substrate for cellular expansion due to its inherent hydrophobicity [40]. Ang et al. [41] similarly reported lower cell density and less stratification of primary rabbit conjunctival cells on untreated-PCL compared to PCL treated with Sodium Hydroxide, which made the material more hydrophilic. For dECM-containing scaffolds, two dissimilar morphologies were apparent. Cells either presented as small and rounded with numerous filopodia that contacted with neighbouring cells, or they appeared larger, flatter, and more elongated. This latter morphology was particularly noticeable within the SIS1% group, though stacking of cells was also visible from SEM images. Corroboration with z-plane views did support instances of two (31.35 ± 13.13%) and three (4.79 ± 2.16%) layers, though the majority of nuclei were held as a monolayer (63.86 ± 15.60%). It should be noted that the total number of cell nuclei counted within the regions of interest was markedly lower (63.53% difference) than for UBM10%. The number of cell nuclei counted for SIS10% was also low compared to UBM10% with a difference of 20.22%. Yet a larger proportion of these cell nuclei were stacked in two (46.91 ± 5.88%), three (18.50 ± 11.33%) or even four (1.13 ± 0.99%) layers, which suggests a greater quantity of SIS triggered an increase in cell proliferation and a tendency to stratify, though further investigation is needed to confirm this. However, incorporation of UBM did lead to a change in cell response, where a greater number of microvilli were observed on these scaffolds (Figure 7). Microvilli are an indication of epithelial cell polarity [42]. Microvilli at the ocular surface help stabilise the tear film and either their complete absence or limited presence has been noted in patients affected by tear film abnormalities and ocular surface diseases [43,44,45]. Both 1% and 10% UBM scaffolds yielded respective total cell nuclei counts of 329 ± 50 and 392 ± 81, and a shift from monolayers to more than three quarters of all nuclei being held in two, three, four, and even five layers. UBM10% demonstrated the greatest presence of cell stratification with 37.02 ± 7.85% representing two nuclei layers, 33.13 ± 9.07% for three layers, 13.48 ± 8.87% for four layers, and 2.57 ± 1.10% for five layers, where 2, 6, and 4 separate instances of 5 nuclei stacked on top of each other were evident in each sample (*n* = 3, 6 regions of interest).

The number of epithelial cell layers varies across the distinct regions of the conjunctiva: typically, 6 in the bulbar, 3 in the fornix, 2–3 in the upper tarsus, and 4–5 in the lower tarsus of the palpebral [3]. With this in mind, these scaffolds appear to provide suitable mimicry of the conjunctiva by supporting a similar degree of stratification. Replication of three or more cell layers was most evident in scaffolds containing UBM. It should be noted, however, that the z-plane view was for cell nuclei only and thus does not take account of the whole cell body. It is therefore likely that stratification may have occurred to a greater extent, with cell cytoplasm (free from the nucleus) covering a larger area and this potentially being positioned directly on top of other cells. For healthy conjunctival epithelial cells in the bulbar region, the relative size ratio of the cell nucleus compared to the cytoplasm has been reported as 1:4 [46]. Furthermore, the culture time at the air/liquid interface was likely insufficient to support development of an established and more mature epithelium. Several research groups have achieved various layers of stratification for HCjE cells grown on biological substrates, including Gipson et al. [24] where 2–3 layers of HCjE cells were achieved following their culture on transwells coated with collagen type I, and Zorn-Kruppa et al. [47] who similarly demonstrated stratification up to 3 layers for HCjE cells when seeded at high density (750,000) on collagen stromal layers and cultured at the air/liquid interface for 6 days. Other groups have demonstrated the benefit of co-culture systems of HCjE cells and fibroblasts to stimulate stratification, for example, García-Posadas et al. [48] observed 3–5 layers of primary conjunctival epithelial cells following air/liquid interface culture on fibrin-hydrogels containing conjunctival fibroblasts. It is therefore promising that our dECM-PCL electrospun scaffolds have demonstrated the ability to support several cell layers without the need of co-cultures, which may be a consequence of these dECM powders providing a more complete biological cocktail of biomolecules (proteins, glycoproteins, proteoglycans, growth factors, chemokines and cytokines [15]), which would not all be present in these singular biomolecular structures. Whether the extent of this stratification could be enhanced, both in terms of coverage and number of layers, with culture of HCjE cells and fibroblasts would be an interesting study, however.

The subtle differences in UBM composition to SIS do appear to have a played role, but overall, the incorporation of dECM to PCL has provided a level of bioactivity that HCjE cells have responded to. Coupled with notable differences to material composition, particularly fibre diameter and tensile properties, these dECM-containing scaffolds warrant further exploration as potential ATMP substrates for conjunctiva replacement and regeneration. In particular, the ability to support mucin-secreting goblet cells is an essential feature that these, or any, potential ATMPs would need to demonstrate.

The subtle differences in UBM composition to SIS do appear to have played a role. A proteomic study on decellularised UBM tissue suggests that the collective grouping of different collagen types (I, II, III, VI, and XIV) coupled with high quantities of proteoglycans, such as perlecan (essential component for epithelial cell basement membrane formation [49] and therefore predominant in conjunctival basement membrane-ECM [50,51]), make them useful biomaterials for promoting cell growth and tissue remodelling and regeneration, and would be advantageous as constructs to support skin remodelling [52]. To the authors’ knowledge, there is no similar proteomics study for SIS, which would allow a better comparison of the composition of these two tissues; however, a study by Lindberg and Badylak [53] reported lyophilised SIS did not support growth and attachment of epidermal cells as well as non-lyophilised SIS and suggested detrimental structural and/or compositional changes had occurred. As a lyophilised powder, this may have been a contributing factor in this study. Whilst further elucidation of UBM and SIS composition is necessary to truly ascertain the biological effects, this study has demonstrated the incorporation of dECM to PCL has provided a level of bioactivity that HCjE cells have responded to. Coupled with notable differences to material composition, particularly fibre diameter and tensile properties, these dECM-containing scaffolds warrant further exploration as potential ATMP substrates for conjunctiva replacement and regeneration. In particular, the ability to support mucin-secreting goblet cells is an essential feature that these, or any, potential ATMPs would need to demonstrate.

## 5. Conclusions

This study aimed to determine the impact of including powders of decellularised tissue matrices with a synthetic polymer to create electrospun fibre scaffolds. Several differences occurred with fibre fabrication (reduced diameter) and their chemical composition (amine presence, uniform distribution) and tensile properties (increased stiffness, yield stress, strength at break, and decreased extension). These scaffolds also influenced the response of HCjE cells, with greater presence of rounded and clustered cells and their stratification up to five layers high. With development and longer-term culture, these dECM-containing PCL scaffolds may lead to the creation of an innovative ATMP for repair and regeneration of damaged conjunctiva.

## Data Availability

The data presented in this study are available on request from the corresponding author.

## Supplementary Materials

The following is available online at https://www.mdpi.com/1999-4923/13/3/318/s1, Figure S1: Scanning electron microscopy images of electrospun poly(ε-caprolactone) scaffolds with the addition of pepsin-solubilised decellularised tissue powder (dECM): (A) 1% small intestinal submucosa (SIS), (B) 10% SIS, (C) 1% urinary bladder matrix (UBM), and (D) 10% UBM. Magnification ×10,000, scale = 5 μm. (E) Measured fibre diameter presented as a violin plot for each group (*n* = 50).
