# Supplementary material for: Material Characterisation and Stratification of Conjunctival Epithelial Cells on Electrospun Poly(ε-Caprolactone) Fibres Loaded with Decellularised Tissue Matrices
Source: Pharmaceutics. 2021 Feb 28;13(3):318. doi: 10.3390/pharmaceutics13030318 (PMC7997349; doi:10.3390/pharmaceutics13030318)
Supplement: Supplementary file 1 [file pharmaceutics-13-00318-s001.pdf]

pharmaceutics

MDPI

Supplementary Materials: Material Characterisation and Stratification of Conjunctival Epithelial Cells on Electrospun Poly(ε-Caprolactone) Fibres Loaded with Decellularised Tissue Matrices

Lucy A. Bosworth, Kyle G. Doherty, James D. Hsuan, Samuel P. Cray, Raechelle A. D'Sa, Catalina Pineda Molina, Stephen F. Badylak and Rachel L. Williams

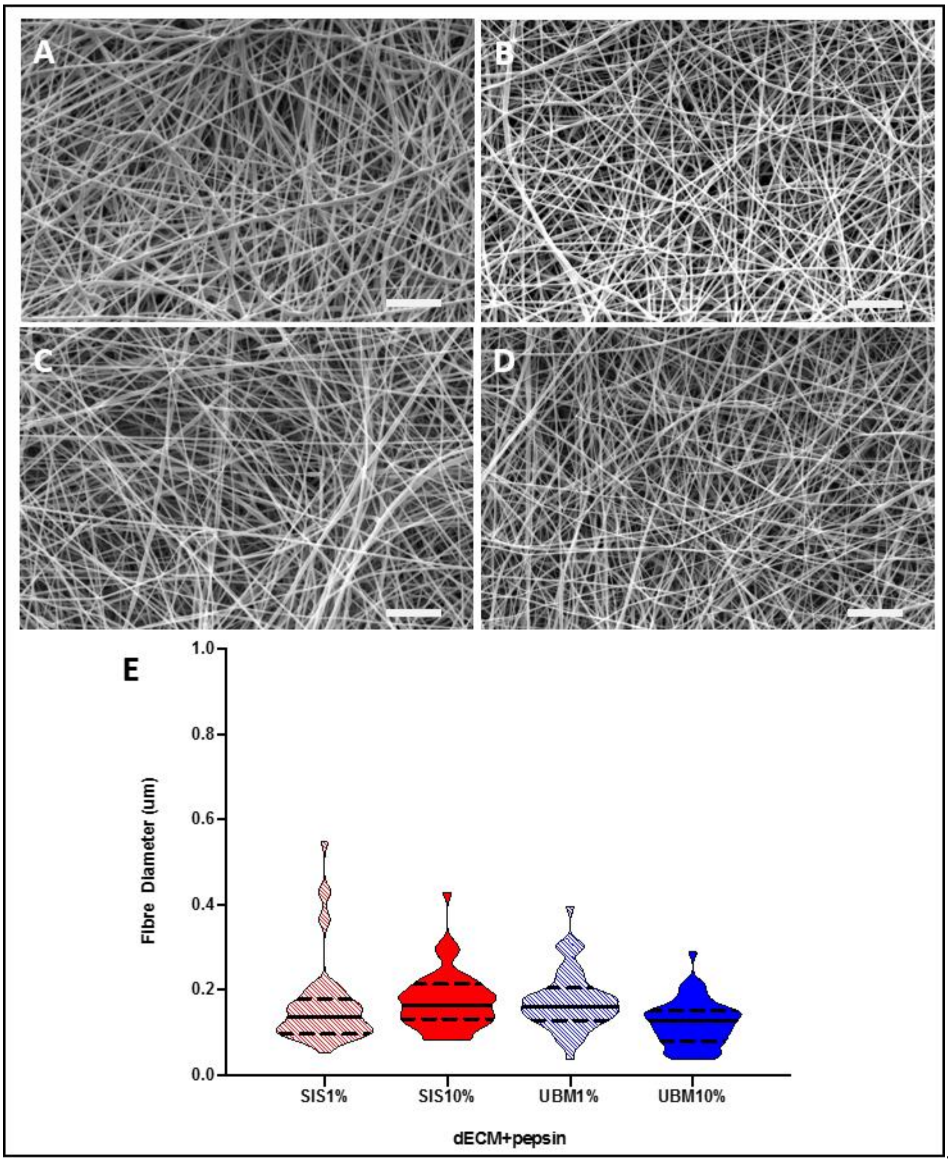

Figure S1. Scanning electron microscopy images of electrospun poly(ε-caprolactone) scaffolds with the addition of pepsin-solubilised decellularised tissue powder (dECM): (**A**) 1% small intestinal submucosa (SIS), (**B**) 10% SIS, (**C**) 1% urinary bladder matrix (UBM), and (**D**) 10% UBM. Magnification ×10,000, scale = 5 µm. (E) Measured fibre diameter presented as a violin plot for each group (n = 50).
